# Long‐term symptoms of polyneuropathy in breast and colorectal cancer patients treated with and without adjuvant chemotherapy

**DOI:** 10.1002/cam4.3129

**Published:** 2020-05-29

**Authors:** Kristine Bennedsgaard, Lise Ventzel, Andreas C. Themistocleous, David L. Bennett, Anders B. Jensen, Anni R. Jensen, Niels T. Andersen, Troels S. Jensen, Hatice Tankisi, Nanna B. Finnerup

**Affiliations:** ^1^ Danish Pain Research Center Department of Clinical Medicine Aarhus University Aarhus Denmark; ^2^ Department of Oncology Aarhus University Hospital Aarhus Denmark; ^3^ Nuffield Department of Clinical Neuroscience University of Oxford Oxford UK; ^4^ Brain Function Research Group School of Physiology Faculty of Health Sciences University of the Witwatersrand Johannesburg South Africa; ^5^ Biostatistics Department of Public Health Aarhus University Aarhus Denmark; ^6^ Department of Neurology Aarhus University Hospital Aarhus Denmark; ^7^ Department of Clinical Neurophysiology Aarhus University Hospital Aarhus Denmark

## Abstract

**Background:**

The aim of this study was to assess chemotherapy‐induced polyneuropathy (CIPN) 5 years after adjuvant chemotherapy in patients with breast and colorectal cancer. The association of CIPN with quality of life, anxiety, and depression was analyzed.

**Methods:**

Of a set of 100 patients with breast cancer and of 74 with colorectal cancer who had undergone surgery and adjuvant chemotherapy in 2011‐2012, 80 and 52 patients alive, respectively, were included together with two reference groups of 249 breast cancer patients and 83 colorectal cancer patients who had undergone surgery only. All patients were sent a questionnaire on alcohol consumption, smoking habits, comorbidity, medicine consumption, and oxaliplatin‐specific questions, as well as the Michigan Neuropathy Screening Instrument questionnaire (MNSIq), the Douleur Neuropathique 4 Questions (DN4q), the EQ‐5D, and the Hospital Anxiety and Depression Scale. Possible polyneuropathy was defined as the presence of numbness and/or tingling in the feet, secondly as a score of ≥4 on the MNSIq. Possible painful polyneuropathy was defined as pain in both feet and a score ≥3 on the DN4q.

**Results:**

The prevalence of possible polyneuropathy defined by numbness and/or tingling in the feet was 38.8% (28.1‐50.3) after adjuvant docetaxel and 57.7% (43.2‐71.3) after adjuvant oxaliplatin, with no significant difference from a previous 1‐year follow‐up (*P* >.35). Fewer had possible polyneuropathy as defined by the MNSIq. Patients with possible polyneuropathy after adjuvant chemotherapy reported significantly lower quality of life than patients treated with surgery only.

**Conclusion:**

Symptoms of polyneuropathy following adjuvant docetaxel and oxaliplatin persist 5 years after treatment and affect quality of life negatively.

## Graphical abstract

Symptoms of polyneuropathy after adjuvant chemotherapy persist after 1 year. Patient with neuropathy has a lower quality of life

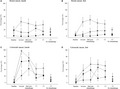

## 1 INTRODUCTION

Chemotherapy‐induced polyneuropathy (CIPN) is one of the most common dose‐limiting side effects and a leading cause of long‐term morbidity and reduced quality of life (QoL) in cancer survivors.^1^, ^2^, ^3^ It is prevalent after treatment with taxanes and platinum agents that are commonly used to treat many types of cancers, including breast, gastrointestinal, lung, ovarian, prostate, and testicular cancer. The most common symptoms are tingling, numbness, and loss of proprioception in a symmetric stocking and glove distribution, whereas neuropathic pain is only seen in a subset of patients.^2^, ^4^, ^5^, ^6^, ^7^ There are no available evidence‐based therapies for the prevention and treatment of CIPN.^8^, ^9^

The prevalence of chronic CIPN after adjuvant treatment with docetaxel and oxaliplatin is considered to be high. Yet, only a few studies have examined the long‐term prevalence and development. Prevalence rates of 36%‐43% of chronic CIPN have been reported up to 3 years after docetaxel treatment.^10^, ^11^, ^12^ A systematic review reported a prevalence of chronic neuropathy between 15% and 79% typically 1‐3 years after oxaliplatin treatment,^13^ and more recent studies have reported prevalence rates between 69% and 79%.^14^, ^15^ Differences in methodology, dropouts, and patient populations may explain some of the variation in prevalence rates. There are no accepted standardized diagnostic criteria for CIPN, and questionnaires are primarily designed to assess the severity of symptoms.^16^ Robust epidemiological data on the long‐term development of neuropathy are needed as most studies use a very rough scale for identifying neuropathy such as the National Cancer Institute – Common toxicity criteria (NCI‐CTC),^17^ have a short follow‐up time, are not prospective,^10^, ^14^, ^18^, ^19^ and do not include a control group.^4^

We have previously reported the development of neuropathic symptoms from baseline up to 1 year after adjuvant docetaxel for breast cancer and oxaliplatin for colorectal cancer.^20^ The current study is 5‐year follow‐up of the same cohort. The aim was to assess the prevalence and severity of symptoms of polyneuropathy and neuropathic pain 4‐5 years after docetaxel and oxaliplatin treatment and to compare these findings with findings from two reference groups treated with surgery only. In addition, we assessed quality of life (QoL), anxiety, and depression to analyze their association with polyneuropathy and painful polyneuropathy.

## 2 METHODS

### 2.1 Patients

All living patients who had participated in a prospective study of patients after surgery and adjuvant docetaxel (n = 100) for high‐risk breast cancer and adjuvant oxaliplatin for high‐risk colorectal cancer (n = 74) at our institution between 2011 and 2012 were included.^20^ In addition, we included two reference groups of patients with the same types of cancer. The reference groups were identified from two Danish registers: the Danish Breast Cancer Group (DBCG)^21^ and the Danish Colorectal Cancer Group (DCCG).^22^

### 2.2 Questionnaires

All participants in the chemotherapy groups who were alive at the 5‐year follow‐up were sent a questionnaire in April 2016. The reference groups were sent a similar questionnaire in June 2017. Both groups received a reminder after 1 month if they did not respond.

Patients filled out a questionnaire including questions about alcohol consumption, smoking habits, comorbidity, and medicine consumption and in addition an oxaliplatin‐specific questionnaire,^23^ the Michigan Neuropathy Screening Instrument questionnaire (MNSIq),^24^ the Douleur Neuropathique 4 Questions (DN4q) questionnaire,^25^ the EQ‐5D,^26^ and the Hospital Anxiety and Depression Scale (HADS).^27^

Possible polyneuropathy was determined at either as the presence of numbness or tingling in the feet based on the oxaliplatin‐specific questionnaire,^23^ or as a score of ≥4/13 on the MNSIq.^24^ We defined possible painful polyneuropathy as pain in both feet and a DN4q score ≥3/7. QoL was assessed using the EQ‐5D.^26^ Anxiety and depression were assessed with the Hospital Anxiety and Depression Scale (HADS).^27^

### 2.3 Ethics

Approval was given by the Danish Data Protection Agency (No. 1‐16‐02‐89‐16 and No. 2011‐4‐5725), the Central Denmark Region Committees on Health Research Ethics (No. 1‐10‐72‐359‐15), and Danish Health and Medicines Authority (No. 3‐3013‐605/1/). The study was carried out in accordance with the Declaration of Helsinki.

### 2.4 Statistical analysis

Means were presented with standard deviation (SD) and medians with a 10%‐90% percentile. Normal distributed data were analyzed with Student's *t* test and otherwise with Mann‐Whitney. Binary data were analyzed with Fisher's exact test. Paired binary data were tested with McNemar's test of no difference with estimates given with an exact 95% CI to assess the change in the presence of neuropathy symptoms from the 1‐year to the 5‐year follow‐up in the patients who had received chemotherapy. Associations were described with odd ratios (OR) with 95% CI, and logistic regression was used to adjust for possible confounders if there were more than five observations. For secondary outcomes, no correction for multiple testing was performed, results are given with prevalence rates and 95% CI. A value of *P *< .05 was considered significant. Statistical analysis was performed with STATA version 14.2.

## 3 RESULTS

### 3.1 Patients and demographics

**Figure 1:**
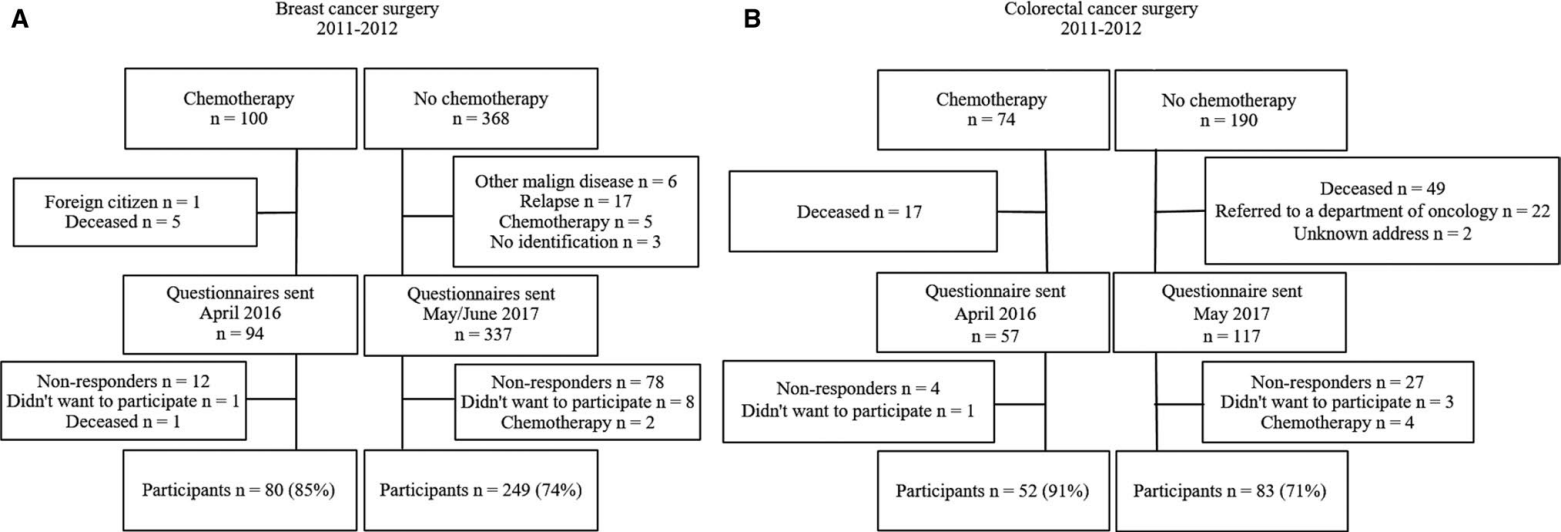
Flowchart of patients who received chemotherapy and their reference groups. In the breast cancer reference group, deceased patients were excluded before transferring the database. A: Breast cancer patients. B: Colorectal cancer patients. Patients were initially excluded from the prospective chemotherapy study if they had metastatic disease, were previously treated with chemotherapy, or could not communicate in Danish. Patients in the reference groups were excluded if they had another known malignant disease, if there was a known cancer relapse or metastatic disease, or if they had received chemotherapy

In the chemotherapy group, 94 (94%) of the original cohort of 100 patients with breast cancer were alive and living in Denmark of which 80 (85%) returned the questionnaire and 57 (77%) of the 74 patients in the colorectal group of which 52 (91%) responded (Figure 1). In the reference groups, 249 of the 337 (74%) surviving patients with breast cancer and 83 of the 117 (71%) eligible patients with colorectal cancer fulfilled the inclusion criteria and answered the questionnaire (Figure 1). The average follow‐up time in the chemotherapy groups was 4.5 (SD, 0.3) years and in the reference group 5.5 (SD, 0.6) years; this will in the following be termed the 5‐year follow‐up.

The two colorectal cancer groups did not differ in gender and age. In contrast, the breast cancer patients in the chemotherapy group were significantly younger than those in the reference group (Table 1). Time since surgery was shorter in both chemotherapy groups than in the reference groups due to the delay in receiving the identification of the patients from the DBCG and DCCG, otherwise, there were no statistically significant differences in baseline characteristics (Table 1).

**Table 1:** Patient Characteristics

	Breast Cancer			Colorectal Cancer		
	Chemotherapy (n = 80)	No Chemotherapy (n = 249)	*P* value	Chemotherapy (n = 52)	No Chemotherapy (n = 83)	*P* value
Age at 5‐year follow‐up, years, mean (SD)	56.6 (7.7)	65.4 (5.0)	<.001	68.0 (8.3)	69.8 (5.2)	.13
Time since diagnosis or surgery, years, mean (SD)	4.4 (0.3)	5.5 (0.6)	<.001	4.6 (0.3)	5.5 (0.6)	<.001
Sex (female), No. (%)	80 (100%)	249 (100%)		19 (36.5%)	31 (37.3%)	.92
Diabetes, No. (%)	3 (3.8%)	25 (10.2%)	.075	5 (9.8%)	7 (8.8%)	.84
Smoker						
Current, No. (%)	11 (14.1%)	36 (14.5%)	.98	9 (17.7%)	7 (8.8%)	.30
Never (less than 100 cigarettes), No. (%)	35 (44.9%)	113 (45.6%)		17 (33.3%)	32 (40.0%)	
Former smoker, No. (%)	32 (41.0%)	99 (39.9%)		25 (49.0%)	41 (51.5%)	
Endocrine therapy at follow‐up, No. (%)	52 (65.0%)	153 (64.6%)*	.94			
Endocrine therapy at baseline, No. (%)	60 (75.0%)	190 (76.6%)	.45			
Radiotherapy, No. (%)	69 (86.3%)	221 (89.5%)	.43	0 (0.0%)	1 (1.3%)*	.41

Abbreviation: SD, standard deviation.

* 6% missing, if nothing else is indicated < 3%.

### 3.2 Prevalence of possible and painful polyneuropathy

In the breast cancer group, 38.8% had tingling or numbness in the feet vs 57.7% in the colorectal cancer group. More patients reported tingling or numbness in the feet than a positive MNSIq (*P* < .001) (Table 2). Significantly more patients in both chemotherapy groups reported tingling/numbness‐defined polyneuropathy than patients in the reference groups, also after correcting for confounders (Table 2). There was a trend toward more frequent MNSIq‐defined polyneuropathy in the chemotherapy groups (Table 2).

**Table 2:** Proportion of Patients with Symptoms of Polyneuropathy and Neuropathic Pain

	Breast cancer					
	Chemotherapy (n = 80)*	No chemotherapy (n = 249)**	OR (CI 95%)	*P* value	aOR(CI 95%)^a^	aP value
Polyneuropathy						
Tingling or numbness in feet, % (CI 9%)	38.8% (28.1‐50.3)	20.2% (15.3‐25.8)	2.5 (1.4‐4.3)	.001	3.2 (1.6‐6.4)	.001
MNSIq ≥4, % (CI 95%)	17.7% (10.0‐27.9)	11.8% (8.1‐16.6)	1.6 (0.8‐3.2)	.18	2.0 (0.8‐3.3)	.10
Neuropathic pain						
Pain in both feet and DN4 ≥3, % (CI 95%)	13.8% (7.1‐23.3)	2.4% (0.9‐5.2)	6.7 (2.4‐18.7)	<.001	6.2 (1.8‐21.9)	.006

	Colorectal cancer					
	Chemotherapy (n = 52)	No chemotherapy (n = 83)**	OR (CI 95%)	*P* value	aOR (ci 95%)^b^	a*P* value
Polyneuropathy						
Tingling or numbness in feet, % (CI 95%)	57.7% (43.2‐71.3)	23.5% (14.8‐34.2)	4.4 (2.10‐9.45)	<.001	4.8 (2.2‐10.4)	<.001
MNSIq ≥4, % (CI 95%)	19.2% (9.6‐32.5)	9.6% (4.3‐18.1)	2.2 (0.81‐6.09)	.12	2.2 (0.80‐6.0)	.11
Neuropathic pain						
Pain in both feet and DN4 ≥3, % (CI 95%)	21.2% (11.1‐34.7)	2.4% (0.3‐8.4)	10.7 (2.3 −51.4)	.003	11.4 (2.4‐54.4)	.002

Abbreviations: CI, confidence interval; DN4, Douleur Neuropathique 4 Questions; MNSI, Michigan Screening Instrument; OR, odds ratio.

^a^ Adjusted for diabetes, age and treatment with endocrine therapy.

^b^ Adjusted for diabetes, age and gender.

* Data missing from 1%‐2%.

** Data missing from 2%‐5%. MNSIq was positive in two of the patients in the chemotherapy and nine patients in the reference group, respectively, where they did not report symptoms of tingling/numbness.

Patients who had received chemotherapy had an higher frequency of possible neuropathic pain in the feet: 13.8% in the breast cancer chemotherapy group vs 2.4% in the reference group and 21.2% in colorectal cancer chemotherapy group vs 2.4% in the reference group (Table 2). The proportion of possible neuropathic pain among those with tingling/numbness‐defined polyneuropathy was 11 out of 31 (35.5% [19.2%‐54.6%]) in the breast cancer chemotherapy group vs 6 out of 48 (12.5% [4.7%‐25.2%]) in the reference group; 10 out of 30 (33.3% [17.3%‐52.8%]) in the colorectal cancer chemotherapy group vs 2 out of 19 (10.5% [1.3%‐33.1%]) in the reference group. Of note, except for one patient, all of the patients with neuropathic pain had polyneuropathy defined by the presence of tingling or numbness.

### 3.3 Symptoms of polyneuropathy

**Figure 2:**
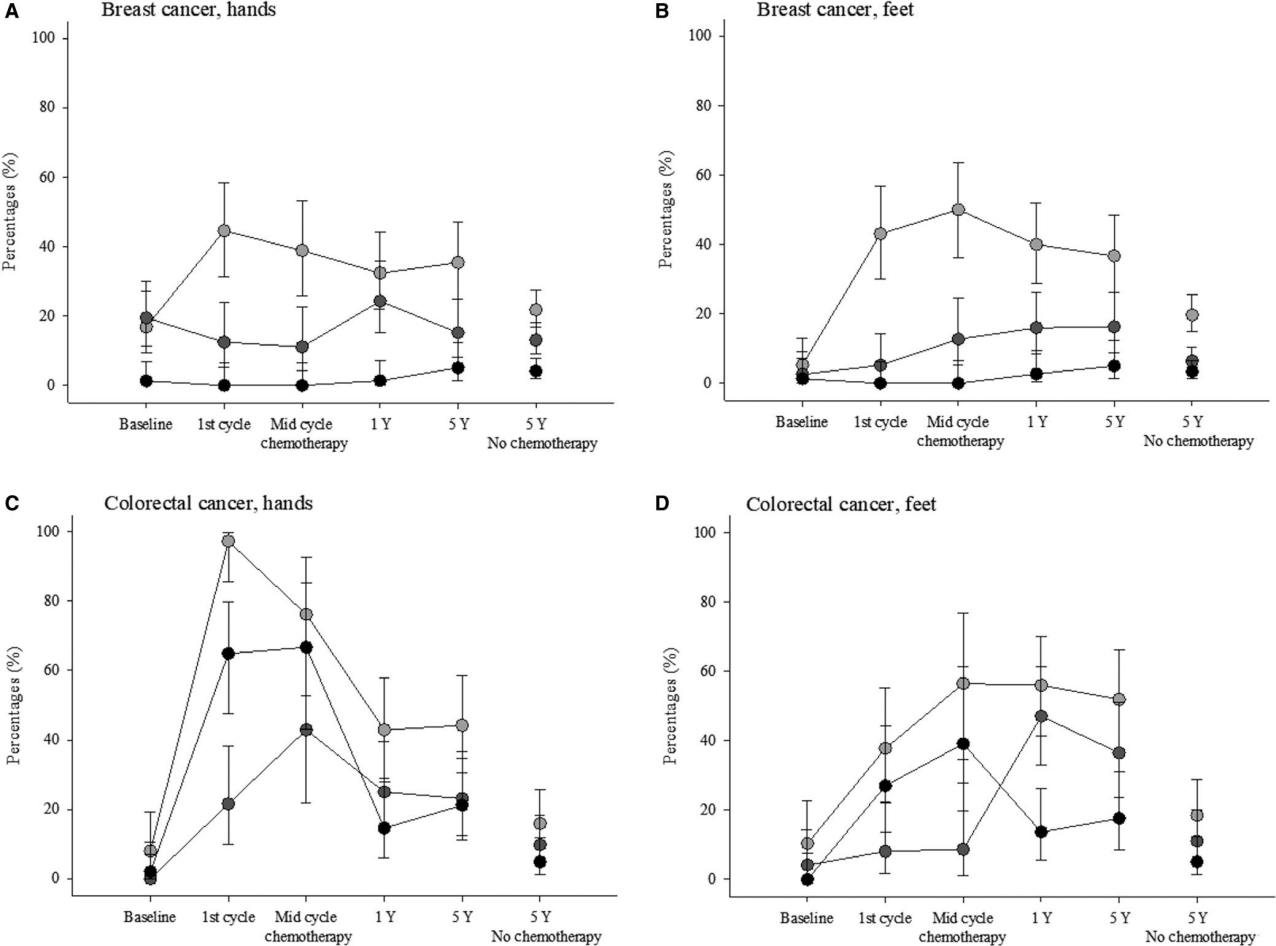
The proportion of patients with neuropathic symptoms in the hands and feet in patients with breast cancer (A and B) and colorectal cancer (C and D). Light gray circle: tingling, dark gray circle: numbness and black circle: burning pain when touching something cold. Data from baseline, during chemotherapy and 1‐year follow‐up questionnaires are included in the chemotherapy groups. Only data from patients who participated at all‐time points are included

The type and severity of the neuropathic symptoms derived from the oxaliplatin‐specific questionnaire^23^ are shown in Table S1. Both chemotherapy groups reported more symptoms in the lower extremities than their reference groups, and in addition patients in the colorectal cancer chemotherapy group reported a higher number of symptoms in the upper extremities than their reference group (Table S1). Burning pain or discomfort with cold, which is a very common symptom in acute oxaliplatin‐induced neuropathy, was still present in 21.2% (11.1%‐34.7%) in the upper extremities and in 17.7% (8.4%‐30.9%) in the lower extremities in patients in the colorectal cancer chemotherapy group, which is significantly higher than in the reference group (Table S1, Figure 2).

### 3.4 Other Pain

Looking at pain in the surgical areas, 24.0% (19.5%‐29.0%) of the patients in the breast cancer group vs 3.7% (1.2%‐8.4%) in the colorectal cancer group experienced pain in the breast/shoulder area. Pain in the gastrointestinal area was experienced by 14.1% (8.7%‐21.1%) of the patients in the colorectal cancer group vs 1.8% (0.7%‐3.9%) in breast cancer group. There were no significant differences in the prevalence of other pain, and pain in the breast/shoulder or in the gastrointestinal areas (data not shown) between the groups who received chemotherapy compared with their reference groups.

### 3.5 Quality of life, anxiety, and depression

No significant differences in the QoL scores between the chemotherapy groups and their reference groups were seen, however, within each group, patients with symptoms of neuropathy had significantly lower QoL scores than those without neuropathy (Table 3). In the breast cancer group, there was a trend toward more anxiety and depression among those who had been treated with chemotherapy (Table 3).

**Table 3:** Quality of Life, Anxiety, and Depression

	Breast cancer							
	Chemotherapy	*P* value	No chemotherapy	*P* value	Estimates (CI 95%)	*P* value	aEstimates (ci 95%)^b^	a*P* value^b^
EQ‐5D								
Problems with								
Mobility, No. (%)	11 (13.8%)	37 (15.2%)		0.88 (0.43‐1.83)	0.75	1.37 (0.57‐3.33)	0.481	
Self‐care, No. (%)	4 (5.0%)	4 (1.7%)		3.11 (0.76‐12.77)	0.11	6.67 (1.08‐41.02)	0.041	
Usual activities, No. (%)	21 (26.3%)	47 (19.5%)		1.47 (0.81‐2.65)	0.20	1.74 (0.83‐3.64)	0.141	
Pain/discomfort, No. (%)	38 (47.5%)	86 (35.3%)		1.66 (1.00‐2.77)	0.051	1.53 (0.82‐2.86)	0.180	
Anxiety/depression, No. (%)	27 (34.2%)	41 (17.1%)		2.52 (1.42‐4.47)	0.002	2.69 (1.33‐5‐46)	0.006	
Quality of life, median (percentiles)	81.5 [70;95]		90.0 [76;97]		‐3.81 (−8.14‐0.51)*	0.084	‐5.33 (−10.50‐ −17.62)*	0.043
With neuropathy^a^	80.0 [45;95]	.011	75.5 [39;94]	<.001				
Without neuropathy^a^	90.0 [59;100]		90.0 [70;100]					
Hospital Anxiety and Depression Scale								
Anxiety score, median (percentiles)	3 [0;10]	3 [0;8]	0.81 (−0.05‐1.66)*	0.066	0.63 (−0.43‐1.70)*	0.24		
Anxiety score ≥ 8, No. (%)	18 (22.8%)	39 (16.1%)	1.54 (0.82‐2.89)	0.18	2.05 (0.94‐4.47)	0.071		
Depression score, median (percentiles)	1 [0;6]	1[0:5]	0.68 (0.08‐1.28)*	0.028	0.71(−0.03‐1.45)*	0.060		
Depression score ≥ 8, No. (%)	7 (8.9%)	7 (2.8%)	3.33 (1.13‐9.82)	0.029	2.69 (0.68‐10.57)	0.16		

	Colorectal Cancer							
	Chemotherapy	*P* value	No Chemotherapy	*P* value	Estimates (CI 95%)	*P* value	aEstimates (CI 95%)^c^	aP value^c^
EQ‐5D								
Problems with								
Mobility, n (%)	7 (13.7%)	9 (11.3%)		1.25 (0.44‐3.61)	0.67	1.27 (0.43‐3.78)	.66	
Self‐care, No. (%)	2 (3.9%)	2 (2.5%)		*1.57 (0.21‐11.5)*	0.66			
Usual Activities, No. (%)	11 (21.6%)	12 (15.4%)^§^		1.51 (0.61‐3.75)	0.37	1.53 (0.61‐3.84)	.36	
Pain/ Discomfort, No. (%)	23 (45.1%)	19 (23.8%)		2.64 (1.24‐5.61)	0.012	3.08 (1.40‐6.76)	.005	
Anxiety/Depression, No. (%)	11 (21.6%)	12 (15.0%)		1.55 (0.63‐3.86)	0.34	1.64 (0.64‐4.19)	.30	
Quality of life, median (percentiles)	85.0 [60;100]		90.0 [60;100]		‐ 1.88 (−8.07‐ 4.31)*	0.55	‐1.78 (−8.26‐4.71)*	.59
With neuropathy^a^	80.0 [50;100]	.017	80.0 [20;95]	]<.001				
Without neuropathy^a^	92.5 [80;100]		92.0 [75;100]					
Hospital Anxiety and Depression Scale								
Anxiety score, median (percentiles)	3 [0;8]	2 [0;8]	0.33 (−0.89‐1.55)*	0.59	0.35 (−0.88‐1.57)*	.58		
Anxiety score ≥ 8, n (%)	6 (11.5%)	8 (10.1%)	1.16 (0.38‐3.55)	0.80	1.01 (0.30‐3.42)	.98		
Depression score, median (percentiles)	1 [0;6]	1 [0;4]	0.06 (−0.79‐0.90)*	0.89	0.02 (−0.86‐0.91)*	.96		
Depression score ≥ 8, n (%)	1 (1.9%)	1 (1.2%)	1.57 (0.10‐25.64)	0.75				

Missing: If nothing else is indicated < 5%, ^§^6% missing.

^a^ Based on the oxaliplatin‐specific questionnaire.

^b^ Adjusted for diabetes, age, and treatment with endocrine therapy.

^c^ Adjusted for diabetes, age, and gender.

* Estimates not being OR (CI 95%).

### 3.6 Follow‐up of patients with chemotherapy

The oxaliplatin‐specific questionnaire^23^ was used at baseline and at the 1‐year and 5‐year follow‐up, and the prevalence of the most common symptoms is presented in Figure 2. When using numbness or tingling in the feet as a marker for polyneuropathy, 7.9% (3.0%‐16.4%) of breast cancer patients had polyneuropathy at baseline, 42.7% (31.3%‐54.6%) at the 1‐year follow‐up, and 38.8% (28.1%‐50.3%) at the 5‐year follow‐up. There was no statistically significant difference in the number of patients with possible polyneuropathy between 1 and 5 years (*P* = .65). Polyneuropathy was present at both time points in 28% of the patients, in 15% at the 5‐year but not the 1‐year follow‐up, and in 11% at the 1‐year but not the 5‐year follow‐up.

In the colorectal cancer group, 14.6% (6.1%‐27.8%) had neuropathy at baseline, 64.7% (50.1%‐77.6%) at the 1‐year follow‐up, and 57.7% (43.2%‐71.3%) at the 5‐year follow‐up. Again, there was no statistically significant difference in the number of patients with possible polyneuropathy between 1 and 5 years (*P* = .34). Polyneuropathy was present at both time points in 51% of the patients, in 6% at the 5‐year but not the 1‐year follow‐up, and in 14% at the 1‐year but not the 5‐year follow‐up.

## 4 DISCUSSION

In this long‐term prospective study including two large reference groups, we could document that polyneuropathy and neuropathic pain were frequent long‐term side effects of docetaxel and oxaliplatin treatment with a negative impact on quality of life. The persistence of CIPN symptoms from the 1‐year to the 5‐year follow‐up suggests that the symptoms become chronic after 1 year.

Our study is one of a few prospective studies with a follow‐up time of 5 years. The finding that symptoms of neuropathy persist for 5 years is important as it suggests that the neuropathy becomes chronic and possibly lifelong. In a shorter term study in patients treated with oxaliplatin, Park et al reported that symptoms and clinical signs of neuropathy and neurophysiological deficits persisted at a 2‐year follow‐up,^28^ and in a small study of 45 patients treated with cisplatin or oxaliplatin, Brouwers et al reported neuropathy symptoms in the feet for up to 6 years.^29^ Another study found the neuropathy to be less severe after 4 years than after 1 year.^30^ Following docetaxel treatment, one study found the symptoms to persist for 13 years after chemotherapy.^10^

The lack of simple reliable diagnostic tools to estimate CIPN is a challenge in epidemiological and clinical studies.^9^ Using the presence of tingling or numbness, which is also used in US National Cancer Institute's Patient‐Reported Outcomes Version of the Common Terminology Criteria for Adverse Events (PRO‐CTCAE),^31^ and in almost every other peripheral neuropathy screening questionnaire,^32^ identified more patients with possible CIPN than using the MNSIq. This is expected since the MNSIq criteria require a score of at least 4/13, of which one was numbness.

Our study showed that it is critical to include a reference group as not all symptoms of neuropathy in the feet are caused by chemotherapy,^20^ emphasizing that the prevalence of chemotherapy‐related symptoms is likely to be overestimated in studies without a reference group. Some symptoms were seen before treatment start. The reasons for the relatively high prevalence of patients with tingling or numbness in the feet at baseline (7%‐15%) and in the reference groups (20%‐24%) are not known. Diabetes is known to give similar symptoms as CIPN,^33^ but only 9.8% of the patients in the reference group in our study had known diabetes. Symptoms reported from the hands in breast cancer patients could most likely be related to surgery, endocrine therapy, radiation therapy lymphedema, or carpal tunnel syndrome,^34^ but we do not have information to look further into that.

We found that approximately one third of patients with symptoms of neuropathy had possible neuropathic pain after oxaliplatin and docetaxel treatment.^25^ This is higher than in a previous prospective study that found that pain in the feet described as burning or aching pain was present in 13% of those who received oxaliplatin 5.5 years ago compared with 6% who did not receive oxaliplatin.^4^ The use of different outcomes measures may explain the difference in the prevalence rates of neuropathic pain. The prevalence of pain in the area of surgery is consistent with other studies.^35^, ^36^

A significantly lower quality of life score was found in the patients who had symptoms of tingling and numbness. This is consistent with earlier studies on CIPN.^37^

Other studies are consistent with our findings with tingling and numbness being the most common symptom.^14^, ^18^ Symptoms of burning pain when touching something cold were still present in the hands and feet in 20% of those who received oxaliplatin at the 5‐year follow‐up. This symptom is very common in the acute phase of chemotherapy.^38^ It is often suggested that the acute symptoms are reversible,^18^, ^39^ but this finding suggests that this may not be the case for a subgroup of patients.

The strength of our study is the long follow‐up, low dropout rate, detailed assessment of neuropathic symptoms, and the inclusion of reference groups. However, the study also has some limitations. As our study is not a randomized trial, we cannot rule out that the higher prevalence of symptoms of neuropathy and pain in the chemotherapy groups is related to other factors, for example, factors related to cancer type or decision to give chemotherapy.

There was a difference in follow‐up periods, but given that both groups were contacted more than 4 years after surgery, we do not expect this to have substantial influence. We did not ask about relapses in the chemotherapy groups, which may have had an impact on the presence and severity of CIPN, as taxanes are used in the treatment of recurrent disease and a high cumulative dose is a known risk factor for developing CIPN.^40^

## 5 CONCLUSION

Symptoms of neuropathy and neuropathic pain were common chronic complications to docetaxel and oxaliplatin treatment. The prevalence of neuropathic symptoms was significantly more common in the groups who received chemotherapy than those who had surgery alone. Symptoms of neuropathy were associated with a lower QoL. Possible neuropathic pain in the feet was found in one third of the patients with symptoms of neuropathy.

## CONFLICT OF INTEREST

NBF has received honoraria for serving on advisory boards from Teva, Novartis, Grünenthal, Mitsubishe Tanabe, Merck, and Novartis and on speaker panel from Astellas. ABJ have received a travel grant from Pfizer and have given presentations at meetings sponsored by Pfizer and Novartis. DLB has acted as a consultant on behalf of Oxford Innovation for Abide, Amgen, Mitsubishi Tanabe, GSK, TEVA, Biogen, Lilly, Orion, and Theranexus. The remaining authors have no conflict of interest.

## AUTHOR CONTRIBUTIONS

Kristine Bennedsgaard: Conceptualization, data curation, formal analysis, investigation, methodology, collection and assembly of data, writing—original draft and editing. Lise Ventzel: Conceptualization, funding acquisition, investigation, methodology, writing—original draft and editing. Andreas C. Themistocleous: Conceptualization, methodology, writing—review draft and editing. David L. Bennett: Conceptualization, funding acquisition, methodology, writing—review draft and editing. Anders B. Jensen: Conceptualization, methodology, writing—review draft and editing. Anni R. Jensen: Conceptualization, methodology, writing—review draft and editing. Niels T. Andersen: formal analysis, writing—review draft and editing. Troels S. Jensen: funding acquisition, writing—review draft and editing. Hatice Tankisi: Conceptualization, methodology, writing—original draft and editing. Nanna B. Finnerup: Conceptualization, formal analysis, methodology, funding acquisition, supervision, writing—original draft and editing.

## TABLE OF CONTENTS

1. Peripheral neuropathy is more common in patients treated with adjuvant docetaxel and oxaliplatin compared to patients treated with surgery only.
2. Symptoms of chemotherapy‐induced polyneuropathy persists between 1 and 5 years after treatment with docetaxel and oxaliplatin, and are associated with lower quality of life in cancer survivors.

## Supporting information

Supplementary Material
