# Supplementary material for: Long‐term symptoms of polyneuropathy in breast and colorectal cancer patients treated with and without adjuvant chemotherapy
Source: Cancer Med. 2020 May 29;9(14):5114–23. doi: 10.1002/cam4.3129 (PMC7367625; doi:10.1002/cam4.3129)
Supplement: Supplementary file 1 — Supplementary Material [file CAM4-9-5114-s001.docx]

**SUPPLEMENTARY TABLE A1. Proportion of Patients with Neuropathic Symptoms and Grading of the Severity of These Symptoms**

|  | Breast Cancer | | | Colorectal Cancer | | | |
| --- | --- | --- | --- | --- | --- | --- | --- |
| A. Upper Extremities | **Chemotherapy,**  **No. (%)** | **No Chemotherapy, No. (%)** | ***P* value** | **Chemotherapy,**  **No. (%)** | **No Chemotherapy, No. (%)** | ***P* value** | |
|  |  |  |  |  |  |  | |
| Tingling | 28 (35.4%) | 52 (21.9%) | **.024** | 23 (44.2%) | 13 (15.9%) | **.001** |  |
| Grading *≥* 3 | 12 (15.2%) | 28 (11.8%) | .44 | 8 (15.4%) | 8 (9.8%) | .41 |  |
| Numbness | 12 (15.2%) | 31 (13.0%) | .71 | 12 (23.1%) | 8 (9.8%) | **.047#** |  |
| Grading *≥* 3 | 5 (6.3%) | 14 (5.9%) | 1.00 | 6 (11.5%) | 4 (4.9%) | .19 |  |
| Difficulty telling the difference between rough and smooth surfaces | 4 (5.1%) | 4 (1.7%) | .11 | 7 (13.7%) | 3 (3.7%) | **.044** |  |
| Grading *≥* 3 | 0 (0.0%) | 0 (0.0%) | 1.00 | 4 (7.8%) | 1 (1.2%) | .07 |  |
| Difficulty feeling hot things | 4 (5.1%) | 1 (0.4%) | **.015** | 6 (11.5%) | 2 (2.4%) | .06 |  |
| Grading *≥* 3 | 2 (2.5%) | 0 (0.0%) | .06 | 2 (3.9%) | 1 (1.2%) | .56 |  |
| Difficulty feeling cold things | 2 (2.6%) | 1 (0.4%) | .15 | 5 (9.8%) | 1 (1.2%) | **.031** |  |
| Grading *≥* 3 | 1 (1.3%) | 0 (0.0%) | .24 | 2 (3.9%) | 0 (0.0%) | .15 |  |
| A greater than normal sense of touch (eg, putting on gloves) | 3 (3.8%) | 7 (2.9%) | .72 | 4 (7.8%) | 1 (1.2%) | .07 |  |
| Grading *≥* 3 | 0 (0.0%) | 5 (2.1%) | .34 | 3 (5.9%) | 0 (0.0%) | .054 |  |
| Burning pain or discomfort without cold | 5 (6.3%) | 10 (4.2%) | .54 | 5 (9.6%) | 4 (4.9%) | .31 |  |
| Grading *≥* 3 | 3 (3.8%) | 10 (4.2%) | 1.00 | 3 (5.8%) | 3 (3.7%) | .68 |  |
| Burning pain or discomfort with cold | 4 (5.1%) | 10 (4.2%) | .76 | 11 (21.2%) | 4 (4.9%) | **.005** |  |
| Grading *≥* 3 | 2 (2.5%) | 8 (3.4%) | 1.00 | 6 (11.5%) | 3 (3.7%) | .089 |  |
| Difficulty identifying objects in your hand (eg, a coin) | 4 (5.1%) | 4 (1.7%) | .11 | 7 (13.5%) | 3 (3.7%) | **.046#** |  |
| Grading *≥* 3 | 1 (1.3%) | 2 (0.8%) | 1.00 | 4 (7.7%) | 1 (1.2%) | .07 |  |
| Do you have involuntary hand movements? | 5 (6.3%) | 6 (2.5%) | .15 | 5 (9.6%) | 5 (6.1%) | .51 |  |
| Grading *≥* 3 | 3 (3.8%) | 3 (1.3%) | .168 | 3 (5.8%) | 2 (2.4%) | *.38* |  |
|  |  |  |  |  |  |  |  |
| Number of symptoms |  |  |  |  |  |  |  |
| 0 | 46 (58.2%) | 172 (72.3%) | .14 | 27 (51.9%) | 66 (80.5%) | **<.001** |  |
| 1 | 16 (20.3%) | 39 (16.4%) |  | 8 (15.4%) | 8 (9.8%) |  | |
| 2 | 11 (13.9%) | 16 (6.7%) |  | 6 (11.5%) | 4 (4.9%) |  | |
| 3 or more | 6 (7.6%) | 11 (4.6%) |  | 11 (21.2%) | 4 (4.9%) |  | |
|  | Breast Cancer | | | Colorectal Cancer | | | |
| B. Lower Extremities | **Chemotherapy,**  **No. (%)** | **No Chemotherapy, No. (%)** | ***P* value** | **Chemotherapy,**  **No. (%)** | **No Chemotherapy, No. (%)** | ***P* value** | |
|  |  |  |  |  |  |  | |
| Tingling | 29 (36.7%) | 47 (19.8%) | **.004** | 27 (51.9%) | 15 (18.5%) | **< .001** | |
| Grading *≥* 3 | 15 (19.0%) | 33 (13.9%) | .28 | 18 (34.6%) | 10 (12.4%) | **.004** | |
| Numbness | 13 (16.3%) | 15 (6.4%)* | **.012** | 19 (36.5%) | 9 (11.1%) | **.001** | |
| Grading *≥* 3 | 6 (7.5%) | 10 (4.3%) | .25 | 11 (21.2%) | 4 (4.9%) | **.009** | |
| Difficulty telling the difference between rough and smooth surfaces | 4 (5.1%) | 1 (0.4%)* | **.015** | 10 (19.6%) | 4 (5.0%) | **.017#** | |
| Grading *≥* 3 | 1 (1.3%) | 1 (0.4%) | .44 | 8 (15.7%) | 2 (2.5%) | **.014** | |
| Difficulty feeling hot things | 4 (5.1%) | 0 (0.0%)* | **.004** | 4 (7.8%) | 2 (2.5%) | .21 | |
| Grading *≥* 3 | 0 (0.0%) | 0 (0.0%) | 1.00 | 1 (2.0%) | 1 (1.3%) | 1.00 | |
| Difficulty feeling cold things | 4 (5.1%) | 0 (0.0%)* | **.004** | 7 (13.7%) | 2 (2.5%) | **.028#** | |
| Grading *≥* 3 | 0 (0.0%) | 0 (0.0%) | 1.00 | 3 (5.9%) | 9 (0.0%) | .06 | |
| A greater than normal sense of touch (eg, putting on socks) | 8 (10.1%) | 12 (5.1%)* | .18 | 10 (19.2%) | 3 (3.8%) | **.006** | |
| Grading *≥* 3 | 6 (7.6%) | 7 (3.0%) | .10 | 9 (17.3%) | 2 (2.5%) | **.007** | |
| Burning pain or discomfort without cold | 6 (7.6%) | 12 (5.1%)* | .41 | 9 (17.7%) | 3 (3.8%) | **.011** | |
| Grading *≥* 3 | 5 (6.3%) | 11 (4.7%) | .56 | 4 (7.8%) | 2 (2.5%) | .21 | |
| Burning pain or discomfort with cold | 4 (5.1%) | 8 (3.4%)* | .51 | 9 (17.7%) | 4 (5.1%) | **.033** | |
| Grading *≥* 3 | 2 (2.5%) | 6 (2.6%) | 1.00 | 6 (11.8%) | 2 (2.5%) | .06 | |
| Legs feeling heavy | 26 (32.9%) | 47 (19.9%) | **.021** | 12 (23.1%) | 13 (16.3%) | .37 | |
| Grading *≥* 3 | 18 (22.8%) | 36 (15.3%) | .17 | 7 (13.5%) | 10 (12.5%) | 1.00 | |
|  |  |  |  |  |  |  | |
| Number of Symptoms |  |  |  |  |  |  | |
| 0 | 37 (46.8%) | 170 (71.4%) | **< .001** | 21 (40.4%) | 60 (73.2%) | **< .001** | |
| 1 | 17 (22.8%) | 31 (13.0%) |  | 7 (13.5%) | 10 (12.2%) |  | |
| 2 | 11 (13.9%) | 18 (7.6%) |  | 8 (15.4% | 7 (8.5%) |  | |
| 3 or more | 13 (16.5%) | 19 (8.0%) |  | 16 (30.8%) | 5 (6.1%) |  | |

Neuropathic symptoms of the upper (A) and lower (B) extremities and grading of severity *≥*3 out of 5 from the oxaliplatin-specific questionnaire. Missing: If nothing else is indicated < 5%, *6% missing. Significant differences (*P* < 0.05) are indicated with bold text (Fisher’s exact test).

#Significant value (*P* < 0.05) changed to non-significant (*P* < 0.05) when patients with diabetes are removed.
